# Alpha band functional connectivity correlates with the performance of brain–machine interfaces to decode real and imagined movements

**DOI:** 10.3389/fnhum.2014.00620

**Published:** 2014-08-08

**Authors:** Hisato Sugata, Masayuki Hirata, Takufumi Yanagisawa, Morris Shayne, Kojiro Matsushita, Tetsu Goto, Shiro Yorifuji, Toshiki Yoshimine

**Affiliations:** ^1^Department of Neurosurgery, Osaka University Medical SchoolSuita, Japan; ^2^Division of Functional Diagnostic Science, Graduate School of Medicine, Osaka UniversitySuita, Japan; ^3^ATR Computational Neuroscience LaboratoriesKyoto, Japan

**Keywords:** brain–machine interfaces, functional connectivity, alpha band, real movement, imagined movement, magnetoencephalography, primary motor area, motor association area

## Abstract

Brain signals recorded from the primary motor cortex (M1) are known to serve a significant role in coding the information brain–machine interfaces (BMIs) need to perform real and imagined movements, and also to form several functional networks with motor association areas. However, whether functional networks between M1 and other brain regions, such as these motor association areas, are related to the performance of BMIs is unclear. To examine the relationship between functional connectivity and performance of BMIs, we analyzed the correlation coefficient between performance of neural decoding and functional connectivity over the whole brain using magnetoencephalography. Ten healthy participants were instructed to execute or imagine three simple right upper limb movements. To decode the movement type, we extracted 40 virtual channels in the left M1 via the beam forming approach, and used them as a decoding feature. In addition, seed-based functional connectivities of activities in the alpha band during real and imagined movements were calculated using imaginary coherence. Seed voxels were set as the same virtual channels in M1. After calculating the imaginary coherence in individuals, the correlation coefficient between decoding accuracy and strength of imaginary coherence was calculated over the whole brain. The significant correlations were distributed mainly to motor association areas for both real and imagined movements. These regions largely overlapped with brain regions that had significant connectivity to M1. Our results suggest that use of the strength of functional connectivity between M1 and motor association areas has the potential to improve the performance of BMIs to perform real and imagined movements.

## INTRODUCTION

The brain signals recorded from the primary motor cortex (M1) are known to serve a significant role in providing the information necessary for brain–machine interfaces (BMIs). This technology is expected to offer patients who have lost control of voluntary movements, including those with amyotrophic lateral sclerosis (ALS) and spinal cord injury, greater independence, and a higher quality of life by enabling them to control external devices to communicate with others and to manipulate their environment at will (Wolpaw et al., 2002; Birbaumer, 2006; Hirata et al., 2012; Hochberg et al., 2012; Collinger et al., 2013). Recently, many studies reported the importance of M1 signals in providing the information necessary for BMIs using various types of signal platforms to execute real and imagined movements; for example, electroencephalography (EEG; Bradberry et al., 2010; Shindo et al., 2011), magnetoencephalography (MEG; Mellinger et al., 2007; Buch et al., 2008; Waldert et al., 2008; Wang et al., 2010; Sugata et al., 2012a), and electrocorticography (ECoG; Leuthardt et al., 2004; Schalk et al., 2007; Yanagisawa et al., 2011, 2012a).

In electrophysiological studies, particular ranges of neural oscillations, which are usually classified into alpha (8–13 Hz), beta (14–25 Hz) and gamma (30–90 Hz), were shown to be associated with motor control (Lopes da Silva, 2013), and their applications to BMIs have been investigated (Wolpaw and McFarland, 2004; Birbaumer, 2006; Yanagisawa et al., 2011). Rhythmic activity in the alpha range observed over the region of the Rolandic fissure is typically not in the form of a sinusoidal curve (Pfurtscheller and Neuper, 1997) and variably referred to as mu rhythm (Gastaut, 1952). It can be observed along with beta band activity during movement (Pfurtscheller and Aranibar, 1977; Cheyne, 2013) and tactile stimulation (Gaetz and Cheyne, 2006). Since modeling of non-sinusoidal waveforms requires the use of higher frequency harmonic components in addition to a fundamental frequency, beta rhythm activity associated with mu rhythm might result from the non-sinusoidal nature of mu rhythms, rather than an independent physiological processes (Jurgens et al., 1995). In addition, the gamma band has been shown to correlate with the firing activities of neurons representing neural information (Ray et al., 2008; Quian Quiroga and Panzeri, 2009). These frequency bands compose task-specific spatial connectivity patterns in movement related neural networks such as those involving the M1, premotor cortex (PMC), and supplementary motor area (SMA; Herz et al., 2012). Among these frequency bands, recently, functional connectivity within the range of alpha band between the sensorimotor area and motor association area was shown to be relevant to post-stroke recovery potential (Westlake et al., 2012). In this study, neural oscillations of the alpha band were used to calculate functional connectivity because of the higher signal-to-noise ratio compared to oscillations in other frequency bands (e.g., theta, beta) and because they play an important role in controlling cortical excitability. In addition, alpha oscillations are also relevant in the controlling of motor execution through the modulation of gamma-band activity (Yanagisawa et al., 2012b). This alpha oscillation in the sensorimotor cortex, i.e., mu rhythm, has been observed in relation to not only motor execution (Salmelin and Hari, 1994; Leocani et al., 2001) but also motor preparation (Pfurtscheller et al., 1997; Pineda, 2005) and motor imagery (Pfurtscheller et al., 2006; Llanos et al., 2013) as well as beta oscillations, and is considered a mechanism for improving information processing during these tasks (Basar et al., 2001; Palva and Palva, 2007; Sabate et al., 2012). Furthermore, functional connectivities within the range of alpha band activity are suggested to be related to physical and mental fitness (Douw et al., 2014). Such neurophysiological aspects have also been proposed as useful markers of impaired brain states, such as schizophrenia (Hinkley et al., 2011), Alzheimer’s disease (Canuet et al., 2012), and multiple sclerosis (Cover et al., 2006). However, in the field of BMIs, there have been few studies focusing on the relationship between functional connectivity within the range of alpha band activities and the performance of BMIs. Based on the above findings, we hypothesized that alpha band activity is a key component to revealing the relationship between the functional connectivity of M1 and performance of BMIs in decoding real and imagined movements. We further hypothesized that brain regions possessing strong alpha functional connectivity with M1 contribute to the performance of BMIs.

The aim of this study was to clarify the relationship between alpha functional connectivity and the performance of BMIs. For this purpose, we used MEG to examine the relationship between the performance of neural decoding, which has been also termed “decoding accuracy” in several studies (Waldert et al., 2008; Bradberry et al., 2010; Yanagisawa et al., 2011), and functional connectivity of activities within the alpha band (8–13 Hz). MEG has several advantages for analyzing functional connectivity compared with EEG and fMRI. MEG has a higher spatial resolution than EEG, and can record a direct correlate of neural activity with high temporal resolution compared with fMRI. We extracted 40 virtual channels in the left M1 using a beam forming approach and used them as a decoding feature. In addition, we calculated seed-based functional connectivity over the whole brain using alpha band activity. Seed voxels corresponded to the same locations as the 40 virtual channels set in the left M1. We then computed the task-related functional connectivity instead of that during the resting state because previous studies using MEG (Bardouille and Boe, 2012) and fMRI (Newton et al., 2007; Treserras et al., 2009) showed that functional connectivity during motor tasks is greater than that in the resting state. After calculating the task-related functional connectivity, the correlation coefficients between decoding accuracy and strength of functional connectivity were calculated over the whole brain.

## MATERIALS AND METHODS

### PARTICIPANTS

Ten healthy volunteers participated in this study (five males and five females; mean age 29.8 ± 13.2 years). All participants were confirmed to be right-handed using the Edinburgh Handedness Inventory (Oldfield, 1971; all participants had a score of 100), had no history of neurological or psychiatric diseases, and had normal vision. The protocol of this study was approved by the ethics committee of Osaka University Hospital and all participants provided informed, written consent.

### TASKS

**FIGURE 1:**
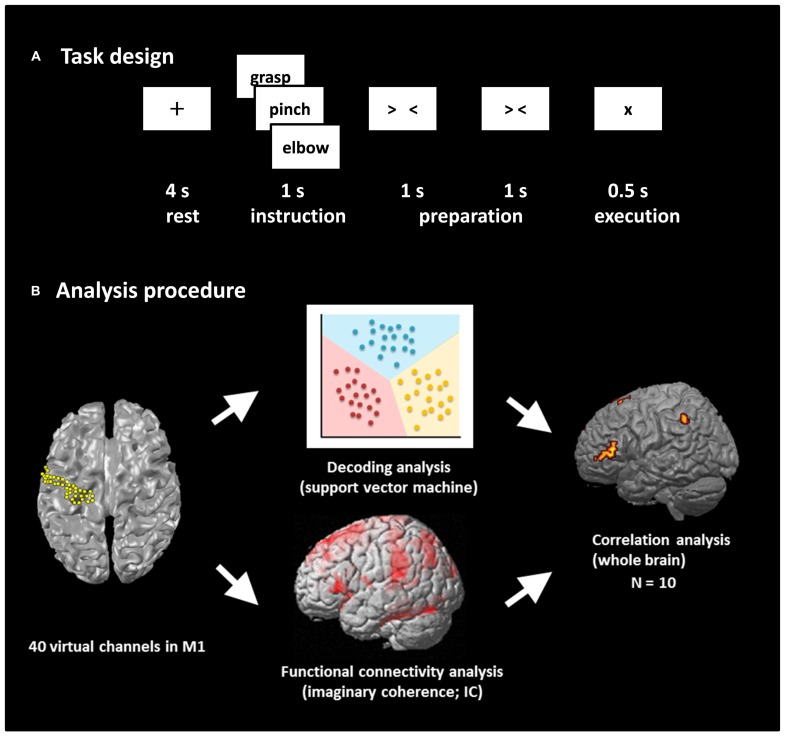
**Task design and analysis procedure. (A)** Task design. Participants performed a real movement task and an imagined movement task following the same task paradigm. Each trial consisted of four phases: a rest phase, an instruction phase, a preparation phase, and an execution phase. In the rest phase, participants fixed their eyes on a black fixation cross “+” presented for 4 s. A Japanese word representing one of three movements was then presented for 1 s during the instruction phase. Then, two timing cues, “> <” and “> <,” were presented during the preparation phase to enable the participants to prepare the execution of real or imagined movements. Finally, the participants performed the movement or imagined performing the movement presented during the instruction phase. Each of the three movements was performed 60 times. **(B)** Analysis procedure. The beam forming approach was used to extract 40 virtual channels from the left M1, and decoding accuracy was calculated using these channels. Seed-based functional connectivity of activities within the alpha band between M1 virtual channels and target voxels over the rest of the whole brain was calculated using imaginary coherence (IC) in individual participants. Then, the correlation coefficient between decoding accuracy and IC was calculated over the participants.

The experimental paradigm is shown in **Figure 1A**. We prepared two tasks: a real movement task and an imagined movement task. We have previously shown the contribution of M1 signals in classifying movement types using these motor tasks based on ECoG (Yanagisawa et al., 2009) and MEG (Sugata et al., 2012b). An epoch started with a 4-s rest phase, and a black fixation cross (+) was presented to fix the participant’s eyes on the screen. Then, a Japanese word representing one of the three right upper limb movements (grasping, pinching, or elbow flexion) was presented for 1 s to instruct the participant which movement to perform or imagine after the appearance of the execution cue. Two timing cues, “> <” and “> <,” were then sequentially presented for 1 s each to enable the participants to prepare the execution of the real or imagined movements. In the real movement task, the participants were instructed to perform the instructed movement presented on the display immediately after the appearance of the execution cue (×). In the imagined movement task, the participants were instructed to imagine performing the movement immediately after the appearance of the execution cue. Each of the three types of movements was performed 60 times during the real movement trials, and the movement in any given epoch was selected randomly. Then the imagined movement trials were conducted in the same manner.

### MEG MEASUREMENTS

Neuromagnetic activity was recorded in a magnetically shielded room using a 160-channel whole-head MEG system equipped with coaxial type gradiometers (MEG vision NEO; Yokogawa Electric Corporation, Kanazawa, Japan). The participant lay on a bed in the supine position with their head centered. The head position was measured before and after recording using five coils placed on the face (the external meatus of each ear and three points on the forehead). Visual stimuli were displayed on a projection screen positioned 325 mm from the participant’s eyes using a visual presentation system (Presentation; Neurobehavioral Systems, Albany, CA, USA) and a liquid crystal projector (LVP-HC6800; Mitsubishi Electric, Tokyo, Japan). Data were sampled at a rate of 1000 Hz with an online low-pass filter at 200 Hz. To reduce contamination from muscle activity and eye movements, we instructed the participants to rest their elbows on a cushion to avoid shoulder movements, and to watch the center of the display without ocular movements and blinking. In addition, to monitor unwanted muscular artifacts, electromyograms (EMG) were simultaneously recorded with electrodes on the *flexor pollicis brevis*, *flexor digitorum superficialis*, and *biceps brachii* muscles during the tasks.

After data acquisition, a 60-Hz notch filter was applied to eliminate the AC line noise, and eye blink artifacts were rejected applying the signal-space projection (SSP), one of the approaches implanted in Brainstorm^1^ to reject external disturbances (Tadel et al., 2011). In addition, to align the MEG data with individual MRI data, three-dimensional data of facial surfaces obtained by laser scanning were superimposed on the anatomical facial surface provided by the individual MRI data with an anatomical accuracy <1 mm.

### VIRTUAL CHANNELS AND PREPROCESSING

To extract M1 signals from the MEG sensor, we used an adaptive, spatial filtering beamforming technique (Sekihara et al., 2002). This approach is used to estimate the temporal course of neural activity at a particular site in the brain marked by an imaging voxel, such as that derived from MRI. The output of such a spatial filter is termed a *virtual channel* or virtual sensor (Robinson and Vrba, 1999). The beamformer is constructed to project signals exclusively from the targeted voxels, while removing residual noise to suppress signals from other parts of the brain. Thus, virtual channels provide data concerning neural activity at target voxels with a considerably higher signal-to-noise ratio than that of raw MEG data (Robinson and Vrba, 1999).

The target location of the virtual channels for the present study was the left M1 gyrus. Forty virtual channels were selected in the M1 with an approximately 2.5 mm inter-sensor spacing using the Montreal Neurological Institute (MNI) coordinates (**Figure 1B**). Then, the virtual channel location coordinates on individual MRIs were extracted utilizing MNI coordinates and warping parameters calculated by Statistical Parametric Mapping 8 (SPM8; Wellcome Department of Imaging Neuroscience, London, UK) using an MRI-T1 template and individual MRI-T1 images. A tomographic reconstruction of the data was created by generating a single-sphere head model based on the head shape obtained from the structural MRIs of each individual participant.

Presentation of the execution cue was defined as the onset of real and imagined movements (0 ms), and all time windows were relative to this time. Epochs from –4000 to 500 ms were analyzed. The baseline was set from –4000 to –3500 ms, during the resting phase. Data from each epoch were normalized by subtracting the mean and then dividing by the SD of the baseline values.

### FUNCTIONAL CONNECTIVITY ANALYSIS

The MEG sensor data were reconstructed in source space with the same beamformer approach described above with 5-mm voxel spacing over the whole brain. The frequency component of the alpha band was chosen to calculate source-space, and seed-based functional connectivity. The functional connectivity at 0–500 ms was calculated with imaginary coherence (IC), one of the connectivity analysis approaches that can reduce overestimation biases in EEG/MEG data generated from common references, cross-talk, and volume conduction (Nolte et al., 2004; Guggisberg et al., 2008; Hinkley et al., 2011). IC rules out real parts of coherence containing similarities with zero time lag, and uses imaginary parts of coherence which contains similarities with a certain time lag, because phase similarities with zero time delay among time series are likely to be caused by crosstalk or volume conduction. Using this method, we can evaluate the “true” interactions between brain areas occurring with a certain time lag. Seed voxels were set at the 40 virtual channels in the left M1 at the same locations described above, and the targets were set as voxels over the remaining whole brain (i.e., except the left M1). The connectivity at each voxel was estimated by averaging across all its Fisher’s Z-transformed connections (Nolte et al., 2004; Guggisberg et al., 2008; Hinkley et al., 2011). All ICs calculated from 40 seed voxels were averaged and used as the strength of functional connectivity between M1 and the target voxel.

Group statistical maps were generated to reveal the brain regions with significant ICs during real and imagined movements. The statistical significance of IC across participants was tested with SPM8. The functional images were normalized using the MNI template in SPM8. A one-sample *t*-test at the voxel level was performed using a *t*-statistic incorporating variance smoothing with an 8-mm Gaussian kernel. Voxels with differences at *p* < 0.01 (familywise error rate, FWER) were considered statistically significant, and were superimposed on the template of the inflated cortical surface brain extracted by FreeSurfer^2^.

### DECODING ANALYSES

Several studies reported that the amplitudes of brain waveforms yield higher performances for BMIs than their power spectrums, such as alpha, beta, and gamma bands (Schalk et al., 2007; Waldert et al., 2008; Yanagisawa et al., 2009). In addition, although the high gamma band activity of ECoG signals is also known to provide high BMI performance (Leuthardt et al., 2004; Yanagisawa et al., 2011), it is difficult for MEG to record high gamma band activity and to obtain high BMI performances. With this in mind, we chose a low frequency component to decode the movement types. The normalized amplitude of the signal recorded at each M1 virtual channel from 0 to 500 ms was resampled over an average 100-ms time window, sliding by 50 ms (9 time points) and then used as a decoding feature. In our preliminary analysis, we also examined other features based on the power spectra of the 40 virtual channels (theta; 4–8 Hz, alpha; 8–13 Hz, beta; 13–25 Hz, low-gamma; 25–50 Hz), but such features did not outperform the normalized amplitudes. Thus, we focused on the decoding results obtained from the low frequency component of the normalized amplitudes.

To examine decoding accuracy, we used a support vector machine (SVM) operating on MATLAB 2013a software (MathWorks, Natick, MA, USA), which was extended to discriminate multiple movements (Kamitani and Tong, 2005; **Figure 1B**). Decoding accuracy was evaluated using 10-fold cross-validation. Each dataset was divided into 10 parts; the classifiers were determined from 90% of the dataset (training set) and tested on the remaining 10% so that the testing dataset was independent from the training dataset for each time point. This procedure was then repeated 10 times. The averaged decoding accuracy over all runs was used as a measure of decoder performance. The binomial test was used to confirm that the decoding performance significantly exceeded chance levels.

### CORRELATION BETWEEN IC AND DECODING ACCURACY

To examine whether functional connectivity is associated with decoding accuracy, we calculated the correlation coefficient between the IC and the decoding accuracy among the ten participants using the Spearman’s rank correlation test over the whole brain (**Figure 1B**). All correlation analyses were corrected for multiple comparisons with a false discovery rate (FDR). Voxels with differences at *p* < 0.05 were considered statistically significant, and were superimposed on the template of the inflated cortical surface brain extracted by FreeSurfer.

## RESULTS

### FUNCTIONAL CONNECTIVITY DURING REAL AND IMAGINED MOVEMENTS

**FIGURE 2:**
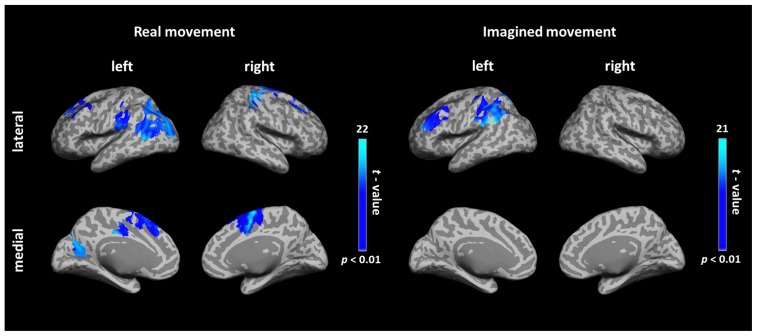
**Cortical connectivity maps during real and imagined movements.** Group (*N* = 10 participants) results of anatomically constrained imaginary coherence (IC) visualized on the inflated cortical surface during real and imagined movements. The brain regions with significant IC with the left M1 are represented in blue (*p* < 0.01, FWER-corrected).

During real movements, statistically significant ICs were observed in the bilateral superior and middle frontal gyri, including the SMA and PMC, in the left parietal lobe and the temporal lobe, and in the right sensorimotor area (**Figure 2** left and **Table 1**). During imagined movements, statistically significant ICs were localized only in the left hemisphere, including the left inferior and superior parietal lobules (IPL, SPL), the superior and middle frontal gyri, and the postcentral gyrus (**Figure 2** right and **Table 2**).

**Table 1:** Brain regions showing significant IC with the left M1 during real movements.

Brain region	Hemisphere	MNI coordinates			*t*-value
		*x*	*y*	*z*	
Superior frontal gyrus	Right	6	-2	70	21.5
Precentral gyrus	Right	24	-19	77	20.5
Cingulate gyrus	Left	-4	-20	42	19.4
Medial frontal gyrus	Left	-2	-6	58	19.2
Precentral gyrus	Right	14	-28	82	18.5
Superior parietal lobule	Left	-36	-70	52	17.6
Middle temporal gyrus	Left	-58	-68	6	17.1
Middle occipital gyrus	Left	-28	-88	16	17.1
Superior temporal gyrus	Left	-50	-30	12	16.0
Superior frontal gyrus	Left	-20	40	40	15.5
Inferior parietal lobule	Left	-38	-56	40	15.5
Angular gyrus	Left	-32	-62	34	15.5
Precuneus	Left	-30	-74	40	15.2
Middle frontal gyrus	Left	-36	30	40	14.5
Superior frontal gyrus	Left	-34	28	50	13.7
Postcentral gyrus	Left	-51	-29	50	11.8

*IC, imaginary coherence; M1, primary motor cortex; MNI, Montreal Neurological Institute template.*

**Table 2:** Brain regions showing significant IC with the left M1 during imagined movements.

Brain region	Hemisphere	MNI coordinates			*t*-value
		*x*	*y*	*z*	
Supramarginal gyrus	Left	-54	-50	28	20.8
Superior temporal gyrus	Left	-52	-32	12	20.3
Inferior parietal lobule	Left	-60	-40	26	18.6
Superior frontal gyrus	Left	-26	56	26	20.6
Superior parietal lobule	Left	-26	-72	64	19.1
Middle frontal gyrus	Left	-44	50	24	17.7
Inferior frontal gyrus	Left	-44	40	16	16.0
Postcentral gyrus	Left	-40	-30	54	10.9

*IC, imaginary coherence; M1, primary motor cortex; MNI, Montreal Neurological Institute template.*

### DECODING ACCURACY FOR REAL AND IMAGINED MOVEMENTS

**FIGURE 3:**
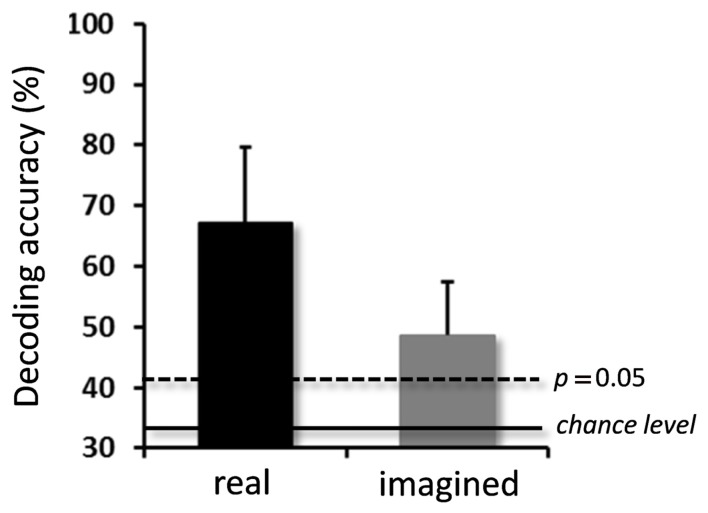
**Averaged decoding accuracies over participants for real and imagined movements.** Significantly high decoding accuracies were obtained for both real and imagined movements (error bar = SD, *N* = 10). The two horizontal lines indicate decoding accuracy at chance level (33.3%, solid line), and at *p* = 0.05 (dashed line, binomial test).

For the real movements, the averaged decoding accuracy among all participants was 67.1 ± 12.5% (mean ± SD), which was significantly higher than chance level (binomial test, *p* < 0.05; **Figure 3**). For the imagined movements, decoding accuracy was also significantly higher than chance level (48.7 ± 8.7%; binomial test, *p* < 0.05), although it was lower than that for the real movements.

### CORRELATION OF IC AND DECODING ACCURACY FOR REAL AND IMAGINED MOVEMENTS

**FIGURE 4:**
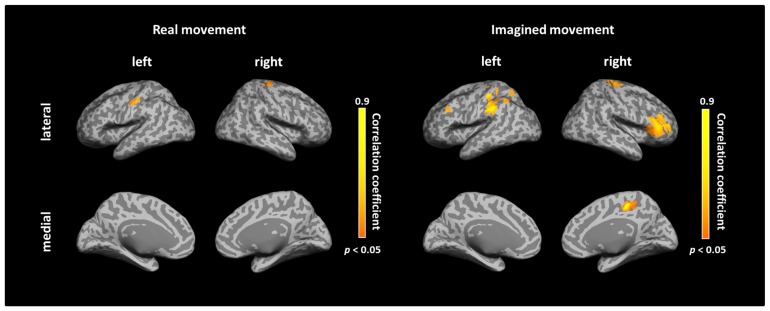
**Spatial distributions of significant correlations between decoding accuracy and connectivity during real and imagined movements.** Correlation coefficients between decoding accuracy and imaginary coherence with the left M1 during real and imagined movements were calculated over the whole brain. Brain regions with significant correlations are represented in orange (*p* < 0.05, FDR-corrected).

After calculating the ICs in individuals, we examined the correlation coefficient between strength of IC and decoding accuracy among all participants during real and imagined movements. **Figure 4** depicts the distribution of significant correlations between IC and decoding accuracy over the whole brain during real movements (*p* < 0.05, FDR-corrected). Significant correlations were localized mainly to the left PMC, postcentral gyrus, and right sensorimotor area (**Figure 4** upper panel and **Table 3**). On the other hand, significant correlations between IC and decoding accuracy for imagined movements were more widely distributed than those of the real movements (**Figure 4** lower panel). In particular, large clusters were observed in the left IPL and SPL and the right inferior frontal gyrus (IFG). Other significant correlations were observed in the left prefrontal cortex (including dorsolateral prefrontal cortex; DLPFC) and right sensorimotor area (**Figure 4** lower panel and **Table 4**).

**Table 3:** Coordinates of significant correlation coefficients between decoding accuracy and strength of IC during real movements.

Brain region	Hemisphere	MNI coordinates			*r*
		*x*	*y*	*z*	
Precentral gyrus	Right	30	-22	74	0.82
Middle frontal gyrus	Left	-38	8	57	0.76
Precentral gyrus	Left	-52	-16	44	0.73
Postcentral gyrus	Left	-50	-20	42	0.71
Postcentral gyrus	Right	24	-30	58	0.69

**Table 4:** Coordinates of significant correlations between decoding accuracy and strength of IC during imagined movements.

Brain region	Hemisphere	MNI coordinates			*r*
		*x*	*y*	*z*	
Postcentral gyrus	Left	-66	-18	30	0.9
Supra marginal gyrus	Left	-66	-22	34	0.9
Inferior parietal lobule	Left	-50	-28	46	0.87
Middle cingulate gyrus	Right	14	-26	46	0.86
Middle frontal gyrus	Right	52	46	8	0.82
Precentral gyrus	Right	26	-22	64	0.81
Inferior frontal gyrus	Right	54	42	8	0.81
Postcentral gyrus	Right	30	-27	70	0.77
Insula	Right	32	24	12	0.76
Middle frontal gyrus	Left	-46	30	34	0.75
Angular gyrus	Left	-40	-58	48	0.75
Superior Parietal lobule	Left	-30	-62	68	0.73

**FIGURE 5:**
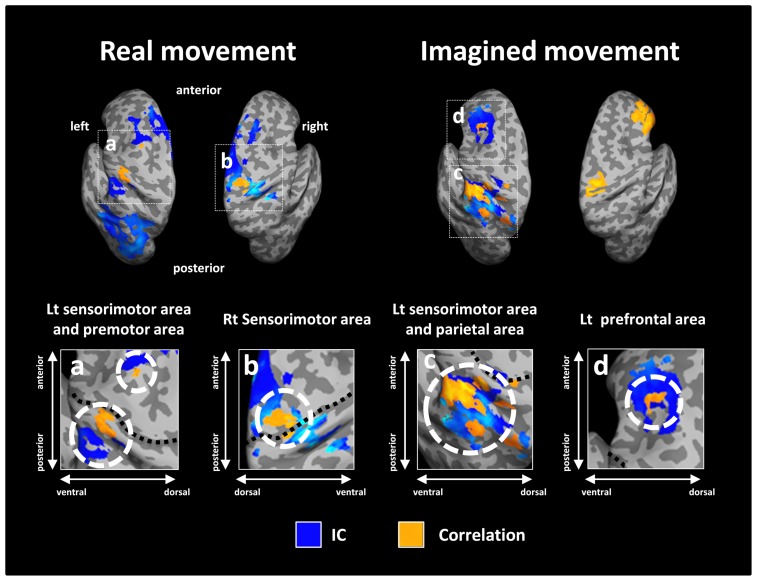
**Overlay of imaginary coherence (IC) and correlation coefficient results for real and imagined movements.** Most of the significant correlations (orange) were located in or around brain regions (white dotted circles) with significant IC (blue). For imagined movements, there was no co-localization between significant correlations and significant IC in the right hemisphere due to the lack of significant IC there. The lower panels indicate magnified figures with brain regions shown using white dotted lines and with corresponding letters on the upper panels. Black dotted lines in the lower panel indicate the central sulcus.

**Figure 5** depicts the overlay map of the distribution of significant correlations between strength of IC and decoding accuracy and significant IC during real and imagined movements. The significant correlations were mainly distributed in or around the brain regions that exhibited significant IC during real and imagined movements. No overlap between correlations and IC was observed in the right hemisphere during imagined movements.

## DISCUSSION

To explore the contribution of functional connectivity to the performance of BMIs, we examined the relationship between neural decoding and alpha band IC with the left M1 during real and imagined movements. The brain regions with significant functional connectivity with M1 during both real and imagined movements were distributed mainly in motor association areas, including the SMA, PMC, and parietal area. In addition, the significant correlations between decoding accuracy and IC strength were distributed in or around the brain regions with significant IC. These results indicate that functional connectivity of alpha band activity between M1 and the motor association area is involved in neural decoding of real and imagined movements.

### FUNCTIONAL CONNECTIVITY IN THE ALPHA BAND DURING REAL AND IMAGINED MOVEMENTS

Recent studies have suggested that fluctuations in alpha band oscillations facilitate processing in task-relevant cortical regions or suppress processing distracting input in task-irrelevant regions to improve task performance (Palva and Palva, 2007; Mazaheri and Jensen, 2010; Gould et al., 2011; Haegens et al., 2011a,b). By holding and releasing high gamma activity during a movement task, we previously demonstrated a functional role of alpha band activity in movement that modulates motor representation in the sensorimotor cortex (Yanagisawa et al., 2012b). Because the main body of alpha band activity recorded over the sensorimotor area is thought to be due to the mu rhythm (Salmelin and Hari, 1994; Leocani et al., 2001), fluctuations in the mu rhythm may play a significant role in controlling the cortical excitability in M1. In fact, it has been demonstrated that data processing improves when the phase of the mu rhythm is modified, and data processing is inhibited when its phase is unlocked (Sabate et al., 2012). Furthermore, the power of the mu rhythm in the sensorimotor area was recently demonstrated to play an important role in cortico-cortical connectivity (Ronnqvist et al., 2013). In the present study, we calculated seed-based functional connectivity from alpha band activity over the whole brain during real and imagined movements. Seed voxels were set as 40 virtual channels in M1. The significant functional connectivity was distributed to the motor association area over frontal and parietal areas during real and imagined movements. The results were mostly concordant with previous studies using fMRI (Solodkin et al., 2004; Gao et al., 2011). Given that these motor association areas have been shown to play important roles in movement planning (Nachev et al., 2008; Andersen and Cui, 2009), movement preparation (Desmurget et al., 2009), and movement intention (Desmurget et al., 2009) by collaborating with M1, the functional connectivity observed in this study may represent the cortical networks of the mu rhythm related to controls of M1 activity during real and imagined movements.

On the other hand, during imagined movements, no significant functional connectivity was observed between the SMA and M1, whereas previous studies reported functional connectivity between these two regions during both imagined and real movements (Solodkin et al., 2004; Chen et al., 2009; Gao et al., 2011). These studies mainly used complex or sequential motor tasks to calculate functional connectivity, while we used three simple right upper limb movements for both real and imagined movements. Because the SMA has been shown to be more sensitive to complex and sequential actions than to simple ones (Nachev et al., 2008), it may be reasonable to expect that we would not observe significant functional connectivity between the SMA and M1 during imagined movements. Furthermore, several studies suggested that the network involved in real movements has a positive influence from SMA on M1, and during imagined movements, the SMA exerts a suppressive influence on M1 (Solodkin et al., 2004; Kasess et al., 2008). These results indicate that the functional connectivity between the SMA and M1 has different characteristics for information processing of real and imagined movements.

### RELATIONSHIP BETWEEN DECODING ACCURACY AND FUNCTIONAL CONNECTIVITY

As described above, the importance of M1 signals for decoding movement types or movement directions has been previously demonstrated using amplitude of waveforms or low frequency components (Waldert et al., 2008; Yanagisawa et al., 2009), sensorimotor rhythm (Mellinger et al., 2007), and high gamma power (Yanagisawa et al., 2012a). An MEG study by Waldert et al. (2008) showed that the low frequency components of neuromagnetic signals are more important for obtaining high decoding accuracy than either alpha-beta or gamma power. We also showed that the fluctuations in the amplitude of low frequency MEG signals carry enough information about hand and arm movements to decode movement kinematics (Sugata et al., 2012b). In the present study, we obtained significantly high decoding accuracy for both real and imagined movements using smoothed M1 signals from 40 virtual channels. Given that the amplitudes of waveforms or the low frequency components of the signals have higher signal-to-noise ratios than the high frequency components, the decoding feature used in this study may be suited for classifying unilateral upper limb movements with single trial MEG signals, even though MEG is inferior to invasive cortical recordings with respect to the sensitivity in weak signals in the high frequency band.

Significant correlations between strength of functional connectivity and decoding accuracy during real movements were observed in motor association areas, such as the left postcentral gyrus and PMC, and the right sensorimotor area. These regions largely overlapped with or were located close to the brain regions with significant IC. Several previous studies reported that the activities of such motor association areas modulate M1 activity by integrating sensory-motor information and transforming the sensory information into motor representation (Luppino and Rizzolatti, 2000; Solodkin et al., 2004; Hoshi and Tanji, 2007; Kantak et al., 2012; Xu et al., 2014). In addition, activity of the PMC during real movement was shown to resemble the activity of M1 neurons, suggesting that the PMC is directly relevant to motor execution (Lee and van Donkelaar, 2006). Thus, the representation of motor information in M1 during real movement may depend on the intensity of sensory-motor integration and activity of the PMC. Furthermore, regarding the correlation in the right sensorimotor area, it is possible that interhemispheric communication between the bilateral M1s, which contribute to controlling the cortico-spinal output from M1 (Avanzino et al., 2007; Lee et al., 2007), was relevant to the representation of motor information in M1.

Significant correlations between functional connectivity and decoding accuracy were observed at the left prefrontal cortex (including DLPFC), IPL, SPL, right IFG, and sensorimotor area during imagined movements. In the left hemisphere, significant correlations around the DLPFC and parietal area overlapped with the brain regions with significant functional connectivity observed in the group analysis. Previous studies showed that the DLPFC and parietal areas were more activated during imagined movement than during real movement (Vry et al., 2012) and play an important role in working memory (Smith and Jonides, 1999; Baddeley, 2003). Motor imagery, which involves simulating movement through the manipulation of visual and kinesthetic information, is a cognitive process that requires working memory (Munzert et al., 2009). Thus, these regions are thought to play a significant role in generating clear motor imagery using working memory. Actually, deactivation of DLPFC was shown to decrease information processing during imagined movement in Parkinson’s disease (Jahanshahi et al., 1995; Samuel et al., 1997, 2001). Furthermore, previous studies reported that the parietal area is associated with accuracy of the imagined movement (Hanakawa et al., 2003) and that lesions to this area reduce motor imagery abilities (Jackson et al., 2001; Lotze and Halsband, 2006; Mulder, 2007). Considering that our results showed that these brain regions are functionally connected with M1 and exhibit significant correlations between decoding accuracy and strength of functional connectivity, it is possible that the DLPFC and parietal area modulate the M1 activity related to the representation of motor information by interacting with the M1 during imagined movement.

In the right hemisphere, significant correlation between decoding accuracy and strength of functional connectivity was observed in the IFG during imagined movement but there was no spatial overlap with significant functional connectivity. The right IFG, but not the left, is relevant to the suppression of movement (Aron et al., 2004; Coxon et al., 2009), and impairment in this region resulted in a loss of suppression of movements in the inhibitory control task (Aron et al., 2004). In addition, a recent study suggested that activation of the right IFG during imagined movement may be relevant to an active inhibition process in the prevention of actual movement (Fleming et al., 2010). Furthermore, the right IFG was reported to be more activated in good imagers than in poor imagers for imagined sequential finger movements (Guillot et al., 2008). More recently, Gaetz et al. (2013) successfully demonstrated that gamma-band activity from the right IFG is observed for tasks involving response interference. Because our results showed that participants with strong functional connectivity between left M1 and right IFG exhibited high decoding accuracy, such inhibition processes in the IFG may work to generate the clear imagery of the movement necessary to decode the imagined movements. Nevertheless, no significant functional connectivity was observed between M1 and IFC in the group analysis, suggesting that connectivity between the two regions is not necessarily required for imagined movement and that the strategy for imagined movement may vary between individuals.

The significant correlation between decoding accuracy and strength of functional connectivity during imagined movement was also distributed to the right sensorimotor area, although there was no spatial overlap with significant functional connectivity. Previous studies reported that the sensorimotor area was activated on not only the contralateral side, but also the ipsilateral side during imagined movement (Kim et al., 1993; Porro et al., 2000). In addition, interhemispheric communication between the bilateral M1s was recently shown during imagined movement as well as real movement, suggesting that bilateral interactions of M1 play a crucial role in the modulation of the motor system during imagined movement (Liang et al., 2014). On the basis of these findings, our results suggest that the strength of functional connectivity between bilateral sensorimotor areas observed in this study may contribute to the modulation of interhemispheric communication between the two regions, and that the subjects with strong connectivity may create more vivid motor imagery related to motor information than the subjects with low connectivity. However, as there was no significant functional connectivity between the bilateral sensorimotor areas in the group analysis, that connectivity may be not an essential component of imagined movement, but rather may be an additive one to generate the M1 activity similar to real movement.

Several studies reported anatomical and functional connectivities between the SMA and M1 (Muakkassa and Strick, 1979; Solodkin et al., 2004; Matsumoto et al., 2007; Nachev et al., 2008). This region was shown to play an important role in movement preparation and movement intention (Lau et al., 2004; Nachev et al., 2008). However, our results showed that there is no significant correlation between decoding accuracy and functional connectivity over the SMA for either real or imagined movements. As described above, the SMA is more sensitive to complex and sequential actions, while the task used in this study was a simple, unilateral, upper limb movement, so that the strength of functional connectivity between M1 and SMA may not have contributed to the decoding performance. It is possible that we can observe the contribution of functional connectivity between M1 and SMA to decoding performance if a more complex task is used. Further studies are needed to clarify this speculation.

### IMPLICATIONS FOR CLINICAL NON-INVASIVE BMIs

To date, many researchers have tried to apply BMIs to patients with severe motor dysfunction using invasive methods, such as ECoG and local field potentials. When we put these invasive BMIs into clinical use, it is indispensable to perform a pre-operative, non-invasive evaluation to determine whether an invasive BMI would be effective. In addition, considering that BMIs are likely to be practically applied to patients with severe motor dysfunction, it is important to improve the performance of decoding accuracy for imagined movements. Recently, several studies reported predictors for the performance of BMIs using sensorimotor rhythm (Blankertz et al., 2010; Hammer et al., 2012), near-infrared spectroscopy activity (Fazli et al., 2012), high theta and low alpha powers (Ahn et al., 2013b), and gamma band activity (Ahn et al., 2013a) during real and imagined movements. In the present study, we focused on the relationship between alpha band functional connectivity and the decoding accuracy of real and imagined movements. As a result, significant correlations between these two aspects were mainly obtained in motor association areas, such as the PMC, sensorimotor areas, and the parietal area. This result suggests that we may be able to predict and improve decoding accuracy by evaluating and enhancing the functional connectivity between M1 and these brain regions, perhaps using neurofeedback methods as previously reported (Shibata et al., 2011; Koush et al., 2013). In particular, for imagined movement, because the strength of functional connectivity observed in this study may be relevant to generation of a vivid imagined movement for decoding movement types, enhancing these networks are important for improving the performance of imagery-based BMIs. Although we used brain signals extracted from the M1 gyrus in the present study, M1 signals from the central sulcus provides a rich source of information representing movement types (Yanagisawa et al., 2009) and have a better signal-to-noise ratio for MEG recordings. They may contribute more to performance of imagery-based BMIs. Also, since the activities of sub-cortical regions as well as the cerebellum are also associated with the generation of voluntary movement (Shibasaki and Hallett, 2006), further investigation of whether functional connectivity between M1 and sub-cortical regions or the cerebellum can be detected and if they are related to the performance of BMIs using this method is thought to be necessary. Additional investigation may lead to the establishment a method for pre-operative evaluation or for the application of the present findings to clinical tools such as neurorehabilitation.

## CONCLUSION

In this study, we examined the relationship between decoding accuracy and alpha functional connectivity during real and imagined movements. The significant correlations between decoding accuracy and the strength of alpha functional connectivity were mainly distributed to motor association areas. Our results indicate that alpha functional connectivity between M1 and the motor association area is important for the improved neural decoding of real and imagined movements. Further investigation may lead to the establishment of a method for pre-operative evaluation or for the application of the present findings to clinical tools such as neurorehabilitation.

## Conflict of Interest Statement

The authors declare that the research was conducted in the absence of any commercial or financial relationships that could be construed as a potential conflict of interest.

## Footnotes

1 http://neuroimage.usc.edu/brainstorm

2 http://freesurfer.net/
